# Regular Is Longer

**DOI:** 10.1177/2041669517728944

**Published:** 2017-09-13

**Authors:** Kyoshiro Sasaki, Yuki Yamada

**Affiliations:** Faculty of Science and Engineering, Waseda University, Tokyo, Japan; Faculty of Arts and Science, Kyushu University, Fukuoka, Japan; Japan Society for the Promotion of Science, Tokyo, Japan; Faculty of Arts and Science, Kyushu University, Fukuoka, Japan

**Keywords:** time perception, pattern regularity, filter-rectify-filter mechanism, bisection, rating

## Abstract

The current study examined whether regularity of dot patterns would influence time perception. We presented observers the dot patterns with three levels of regularity (high, middle and low) and measured the perceived duration of each pattern by bisection and rating methods. The results revealed that the perceived duration of high regular patterns was longer than that of middle and low regular patterns. Thus, we found that stimulus regularity is one of the factors that influence time perception.

Time perception has been underpinned by a multi-source mechanism. So, it has been found that numerous factors affect reproduction, judgement and bisection of the perceived duration of a stimulus (Grondin, 2010). For example, size (Ono & Kawahara, 2007), luminance (Brigner, 1986), motion (Brown, 1995), number of changes (Fraisse, 1963), numerosity (Chang, Tzeng, Hung, & Wu, 2011) and complexity (Schiffman & Bobko, 1974) of a stimulus have been tested as modulators of time perception. However, researchers have not addressed the effect of regularity of a stimulus. Here, we have endeavoured to clarify that regularity can be a new modulator of time perception.

There are several conceptual models of time perception. One of them is the storage-size model (Ornstein, 1969). In this model, the perceived duration of a stimulus is based on amount of information of stimulus: When the stimulus has large amount of information, its perceived duration is longer. Conversely, according to another hypothetical model of time perception, the perceived duration is determined by a neural energy required to encode stimulus (Eagleman & Pariyadath, 2009). To be precise, higher total energy produces a longer perceived duration. Briefly, the perceived duration is assumed to be based on the amount of information or neural energy.

In the pattern perception, the objective index of pattern regularity (randomness) is pattern entropy (Sheth, Nijhawan, & Shimojo, 2000), which indicates the amount of stimulus information. Because pattern entropy is large as the stimulus complexity increases, regular patterns have less pattern entropy than random patterns. Conversely, recent studies have shown that stimulus regularity is processed by a dedicated visual system. This was mainly revealed by adaptation studies (Ouhnana, Bell, Solomon, & Kingdom, 2013; Yamada, Kawabe, & Miyazaki, 2013). After a prolonged exposure to a regular dot pattern caused negative after-effect, another dot pattern was perceived more randomly. The previous studies suggested that a filter-rectify-filter (FRF) mechanism works on the regularity processing. At the first stage, linear filters detect local luminance-defined orientation and spatial frequency. The second stage rectifies the outputs from the first-order filters. At the third stage, the rectified outputs from the second stage are summed by orientation-selective second-order filters, which have larger receptive fields than the first-order filters. The signals of second-order orientation involve pattern regularity perception and regular patterns have more second-order orientation signals than random patterns.

Considering the previous findings of time perception and pattern perception, there were two conflicting hypotheses in addressing the effect of pattern regularity on time perception. On one hand, the storage-size model predicted that the perceived duration of regular patterns would be shorter than that of random patterns because regular patterns have less pattern entropy. On the other hand, based on the neural energy model, regular patterns have more second-order orientation signals, and thus, the perceived duration of regular patterns would be longer than that of random patterns. Thus, we measured the perceived duration of high regular and low regular patterns by two methods: a temporal bisection task (Experiment 1) and a rating task (Experiment 2).

## Results

**Figure 1.**
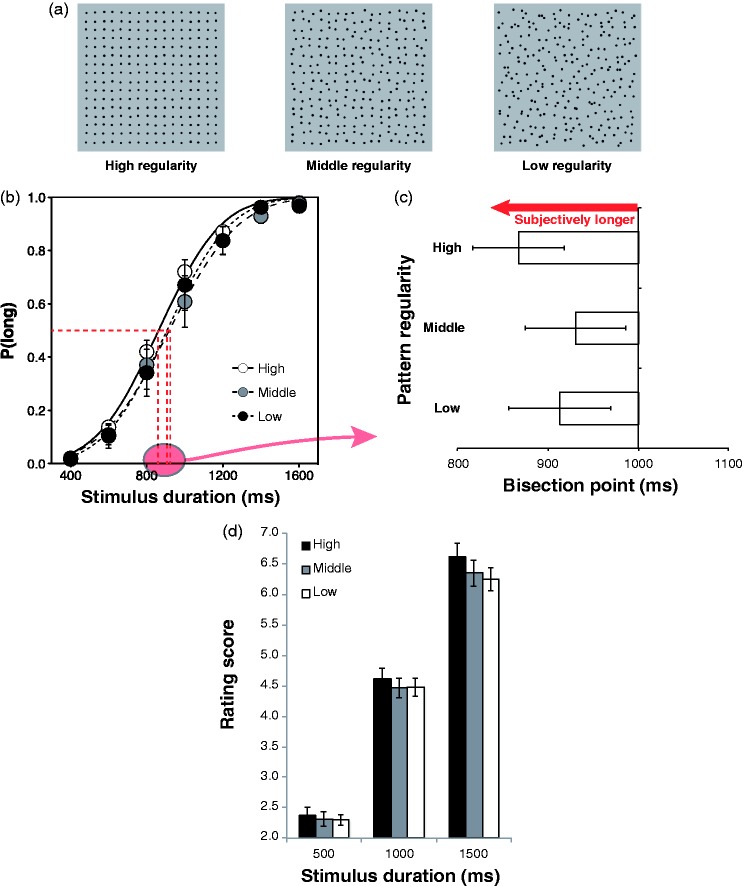
(a) Stimuli used in the experiment. (b) Proportions of long responses as a function of stimulus duration for each level of regularity. Error bars denote the standard errors of the mean. (c) Mean bisection points in each level of regularity. Error bars denote the standard errors of the mean. (d) Mean rating score in each level of regularity and each of stimulus duration. Error bars denote the standard errors of the mean.

We measured perceived duration of high, middle and low regular patterns by the temporal bisection task in Experiment 1. In the learning session, observers learnt two kinds of durations as short and long stimuli. Next, in the judgement session, they observed each pattern with one of the seven durations (see Methods section for details) and judged whether the stimulus duration was perceived as a short or long stimulus. The results are shown in Figure 1(b) and (c). We calculated the proportion of “long” responses on each of the stimulus durations. Then, we also calculated the bisection point of each test stimulus, wherein the proportion of long responses is 50%, by fitting a cumulative Gaussian function to the proportion of long responses as a function of the stimulus duration. Shorter bisection point indicates longer perceived duration of the stimulus. We conducted a one-way analysis of variance (ANOVA) on the bisection point with the pattern regularity as a within-participant factor, and a significant main effect was found, *F*(2, 22) = 10.495, *p* < .001, $\eta_{p}^{2}$^ ^= .49. Multiple comparisons using Holm’s (1979) method revealed that the bisection point was shorter in the high-regularity condition than in the middle, *t*(11) = 3.98, *p* < .007, Cohen’s *d* = 0.34, and low-regularity conditions, *t*(11) = 3.44, *p* < .02, Cohen’s *d* = 0.25. Moreover, we tested differences among three psychometric functions by comparing the bootstrapped 95% confidence interval of the bisection points. As a result, the bisection point was shorter in the high-regularity condition than in the middle regularity (*p* < .01) and in the low-regularity (*p* < .05) conditions.

In Experiment 2, we used the rating method to measure the perceived duration of the pattern stimuli. Stimulus durations were 500, 1,000 and 1,500 ms. Participants were asked to rate the perceived duration of each stimulus with 9-point scale (1: *extremely short*; 9: *extremely long*). We conducted a two-way ANOVA on the rating score with the pattern regularity and the stimulus duration as within-participant factors, and both of the main effects were significant—pattern regularity: *F*(2, 38) = 8.219, *p* = .001, $\eta_{p}^{2}$^ ^= .30; stimulus duration: *F*(2, 38) = 347.324, *p* < .001, $\eta_{p}^{2}$^ ^= .95. The interaction between the pattern regularity and the stimulus duration was not significant, *F*(4, 76) = 1.68, *p* > .16, $\eta_{p}^{2}$^ ^= .08. Multiple comparisons using Holm’s (1979) method revealed that the rating score was larger in the high-regularity condition than in the middle-, *t*(19) = 3.39, *p* = .025, Cohen’s *d* = 0.26, and low-regularity conditions, *t*(19) = 3.96, *p* = .017, Cohen’s *d* = 0.31.

## Discussion

The results suggest that stimulus regularity did affect the time perception. Regular patterns were perceived longer. This finding is consistent with the neural energy model (Eagleman & Pariyadath, 2009): The perceived duration of dot pattern depends on the total amount of second-order orientation signals processed in observing the dot pattern. Our simple observation opens a new avenue for understanding more precisely how time perception is formed by information integration of many stimulus features.

In the previous study involving with the neural energy model, Eagleman and Pariyadath (2009) mainly used sequential stimuli and manipulated the repetition and expectation. Recently, Cai, Eagleman, and Ma (2015) presented several kinds of the stimulus sequences and measured the perceived duration of the last stimulus of the sequence. They also controlled the high-level expectation of the last stimulus by manipulating the probability of the stimulus sequence, regularity of the preceding stimuli in the sequence or whether the last stimulus broke the overlearned sequence. Cai et al. found that the last stimulus in the sequence was perceived as short when the last stimulus and the stimulus before the last one were same. However, if the last stimulus was highly expected, the reduction in the perceived duration of the last stimulus would not be found. Similar results were obtained in the study of Matthews (2015). He consecutively presented two images and asked the participants which of them is perceived as longer. Matthews revealed that the perceived duration of the second stimulus decreased in the repetition sequence (i.e., the first and second stimuli were same) when the repetition sequence comprised 25% or 50% of all the trials. However, when the occurrence ratio of the repetition sequence was high, the reduction in the perceived duration disappeared (rather, sometimes the second stimulus was perceived as longer). These results suggest that the reduction in the perceived duration is triggered by the repetition of the stimulus not by the high-level expectation. Based on this suggestion, it is possible that as the neural energy model assumed, the repetition of the stimulus decreases the neural energy required to process the stimulus, and this amount of the energy determines the perceived duration of the stimulus. Conversely, the high-level expectation might be a higher order factor after coding. Since our stimuli were not sequential and the occurrence ratio was equal among the three types of the stimulus, the high-level expectation should not contribute to our results, and hence, the difference in the perceived duration is likely to be based on the neural energy required to process the pattern stimuli.

The present study found that temporal distortion was induced by pattern regularity. However, it was unclear whether this distortion reflected a perceptual or non-perceptual (e.g., decisional: Yates, Loetscher, & Nicholls, 2012) bias. We used the bisection (Experiment 1) and rating (Experiment 2) methods, and the decisional bias might involve in both the methods. Using another method (e.g., equality judgement) would clarify whether the temporal distortion in the present study stemmed from the perceptual or decisional bias.

Several studies of time perception addressed multiple objects as stimuli. Some of them explored the effect of numerical magnitude on time perception (e.g., Hayashi, Valli, & Carlson, 2013; Xuan, Zhang, He, & Chen, 2007). Other studies investigated how the perceived duration of a target stimulus was modulated by the other stimuli surrounding the target (Ayhan, Revina, Bruno, & Johnston, 2012; Cai & Eagleman, 2015). In addition to these findings, we newly discovered that spatial patterns of the multiple objects on time perception. Of course, because we equalized the number of objects and the measure of the perceived duration of the whole of the pattern stimulus (not one of components), these effects reported in the previous study were less likely to mediate in our results.

The results were inconsistent with the account of the storage-size model. However, this does not necessarily reject the storage-size model as a time perception model. Considering various factors that modulate the perceived duration, time perception is possibly based on a multi-source mechanism. Thus, it was no wonder that either the neural energy or the entropy might be the source of the perceived duration. Then, why was the perceived duration grounded on pattern regularity in the present study? Although it is still unclear, perhaps the neural energy is a weighed source of the perceived duration of the stimulus in comparison with the entropy. Further investigations are warranted for this issue in the future.

Which neural sites are involved with the present phenomenon? The previous study (Yamada et al., 2013) speculated that the lateral occipital complex (LOC), whose neurons are related to the spatial integration of orientation signals (Kourtzi, Tolias, Altmann, Augath, & Logothetis, 2003; Lerner, Hendler, & Malach, 2002), was involved with process of pattern regularity (randomness) on the basis of their psychophysical experiments. Recently, this speculation was supported by a functional magnetic resonance imaging study (Yamada et al., 2017). Together with the neural energy hypothesis, the activity in LOC would be higher when the participants see a regular pattern than a random pattern, and the total amount of second-order orientation signals processed in observing the dot pattern would also be different between them. On the basis of this neural activity difference in LOC, the perceived duration of the dot pattern was possibly determined. Conversely, the first and second stages of the FRF process engage in local processing of each dot and the neural activity of them, respectively. Thus, the neural activities at the first and second stages of the FRF process are unlikely to be different as long as the number of dots and the luminance contrast are same among the three types of the pattern stimuli. Hence, the neural activity stemming from the processing in the first and second stages of the FRF process should not contribute to the temporal distortion in the present study. Future neurophysiological studies would provide direct evidences supporting this idea.

## Methods

### Experiment 1

#### Observers

We recruited observers from the students in our university. As a result, 12 observers participated in the experiment conducted in a dark room. They all were unaware of the purpose of the experiment.

#### Stimuli and procedure

Stimuli consisted of 16 × 16 black dot patterns with three levels of regularity (Figure 1(a)). As in the study of Yamada et al. (2013), stimulus randomness was determined by ω of a uniform function (ω = 1, 4 and 7 for high, middle and low regularities, respectively). Seven stimulus durations were employed (from 400 ms to 1,600 ms by 200 ms).

In the learning session, the training stimulus (a black square) was presented for 400 ms (short) and 1,600 ms (long). The observers were asked to judge whether the duration of the training stimulus was short or long in each trial. They performed 10 trials in the training session. Next, in the judgement session, they judged whether each pattern with one of the seven durations was perceived as a short or long stimulus of the training session. Hence, 420 trials involved 3 Regularity × 7 Duration × 20 Repetitions.

### Experiment 2

#### Observers

We recruited observers from the students in our university. As a result, 20 observers participated in the experiment conducted in a dark room. They all were unaware of the purpose of the experiment and did not participate in Experiment 1.

#### Stimuli and procedure

Although stimuli were identical to Experiment 1, stimulus durations were 500, 1,000 and 1,500 ms. Participants were asked to rate the perceived duration of each stimulus with a 9-point scale (1: *extremely short*; 9: *extremely long*). One hundred and eighty trials involved 3 Regularity × 3 Duration × 20 Repetitions.
